# A Single-Center Experience of Correlation of Pulse Pressure to Mortality of Stroke Hemorrhage Patients in Indonesia

**DOI:** 10.1155/2023/5517493

**Published:** 2023-08-09

**Authors:** Feda Anisah Makkiyah, Saraah Dicha, Rahmah Hida Nurrizzka

**Affiliations:** ^1^Department of Neurosurgery, Universitas Pembangunan Nasional Veteran Jakarta, Jalan RS Fatmawati No. 1 Pondok Labu, Jakarta 12520, Indonesia; ^2^Faculty of Public Health, Universitas Islam Negeri Syarif Hidayatullah Jakarta, Jakarta Selatan, Indonesia

## Abstract

**Introduction:**

The relationship between pulse pressure and mortality in acute stroke hemorrhage patients is a subject of debate. To investigate this relationship in the Indonesian context, a study was conducted due to the increasing prevalence of stroke in the country.

**Methods:**

The study sample consisted of 111 patients with acute stroke hemorrhage admitted to the hospital between January 1, 2016, and December 31, 2019. Patients with sepsis, cancer, or other hematology disorders were excluded, as were those who were lost to follow-up. Statistical analysis was performed using SPSS 22, and correlations were evaluated between various patient characteristics and laboratory values.

**Results:**

It was revealed that patients with a wider pulse pressure were more likely to die (adjusted odds ratio = 3,070) than those with a normal or constricted pulse pressure.

**Conclusion:**

Pulse pressure had an impact on the mortality of patients with acute hemorrhagic stroke.

## 1. Introduction

Stroke is the second highest cause of mortality and third highest cause of disability globally [1]. Over the past 40 years, there has been an increase in the occurrence of stroke in developing nations, while the opposite trend is observed in developed countries [2]. From 2013 to 2018, the incidence of stroke in Indonesia increased dramatically from 7 to 10.7 per 1000 population [3]. Hemorrhagic stroke, caused by ruptured vessel walls, accounts for 15% of all acute strokes and has a higher mortality rate than ischemic stroke. When discussing the pressure within blood vessels, it is important to note the difference between systolic and diastolic pressures, which is known as pulse pressure. As individuals age, the walls of their larger arteries become less pliable due to changes in the vessel walls. Specifically, there is less elastin and more collagen, causing vessels to become more calcified and less flexible. This rigidity creates resistance when the left ventricle contracts, leading to an increase in both systolic and diastolic pressures. It is worth noting that pulse pressure has a clinical correlation with the mortality rates of conditions such as sepsis, cardiovascular diseases, and ischemic stroke, which can exacerbate other health issues [4–6]. There were inconsistencies in the research regarding the correlation between low and high pulse pressure and the resulting outcome [7, 8]. This investigation aims to thoroughly examine the correlation between pulse pressure and acute stroke hemorrhage outcomes in Indonesia. The lack of research on this topic in Indonesia is unacceptable, and this study will provide valuable contributions to the existing knowledge on the matter. It is imperative that we understand the relationship between pulse pressure and acute stroke bleeding outcomes in Indonesia.

## 2. Methods

The participants in this study were patients with acute stroke hemorrhage who were admitted to Cileungsi district hospital between January 1st, 2016, and December 31st, 2019. Patients with sepsis, cancer, other hematological disorders, or those who were lost to follow-up were excluded. The sample size was determined using the Lemeshow 2 proportion hypothesis and nonprobability consecutive sampling method.

Upon admission, the patients' laboratory values and characteristics such as sex, GCS, and systolic and diastolic blood pressure were recorded. Patients were considered to have hypertension if they had a history of hypertension or if their average SBP was greater than 140 mmHg or their DBP was greater than 90 mmHg. Patients were considered to have diabetes mellitus if their fasting blood glucose level was greater than 120 mg/dL or if they were taking oral antidiabetic medication or insulin.

The nurses recorded the patients' BP upon admission to the emergency ward. After the patients had been resting for more than 5 minutes, their SBP and DBP were measured using a blood pressure monitor. The difference between SBP and DBP is known as pulse pressure (PP). A PP of more than 60 mmHg is considered high, while a PP of 60 mmHg or less is considered normal or low. Mortality was defined as patients who passed away while in the hospital. Ethical approval came from the UPN Veteran Jakarta Ethical Committee under 153/III/2021/KEPK.

### 2.1. Statistical Analysis

The patient data include the total number of patients as a percentage and their mean value with standard deviation. The unfavorable outcomes for hemorrhagic stroke in patients with different pulse pressures are represented as percentages on a histogram in the univariable analysis. Statistical analysis was conducted using SPSS 22, and correlations between age, sex, risk factors, GCS, mean HR, SBP max, DBP max, pulse pressure, mean glucose, leucocyte, platelet, and creatinine were analyzed using the chi square test. A significance level of 0.05 was used.

## 3. Results

**Figure 1:**
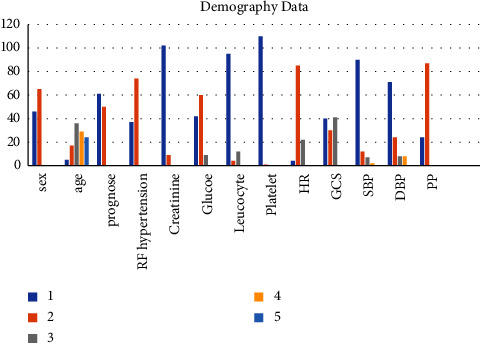
Characteristics of respondents. Sex (1 = women; 2 = men), age (1 = 26–35 years old; 2 = 36–45 years old; 3 = 46–55 years old; 4 = 56–65 years old), prognose (1 = alive; 2 = dead), RF hypertension (1 = none; 2 = yes), creatinine (1 = normal; 2 = abnormal), random blood glucose (1 = <180; 2 = >181–200; 3 = >301), total leucocyte count (1 = <10000; 2 = >10001–<20000; 3 = >20000), total platelet count (1 = >0–<50000; 2 = >50001), HR (1 = <60; 2 = >61–100; 3 = >100), GCS (1 = 3–<8; 2 = 9–13; 3 = 14-15), SBP (1 = >160; 2 = >140–≤159; 3 = >120–<139; 4 = <120), DBP (1 = >100; 2 = >90–<99; 3 = >80–<89; 4 = <80), and PP1, PP2 (1 = <60 normal and narrow; 2 = >61 widen).

From 2016 to 2019, Cileungsi district hospital admitted 123 patients with stroke hemorrhage, excluding 12 respondents (mean age of patients was 54.81 ± 12.865 years; 45.1% were females). The majority of patients were aged 65 years, with more males than females. Most of the patients had grade 2 hypertension with intracerebral hemorrhage but no intraventricular hemorrhage. Their Glasgow Coma Scale (GCS) ranged between 3 and 8, and their heart rates were between 60 and 100 beats per minute, with a mean arterial pressure (MAP) greater than 100 mmHg. Out of 123 patients, 61 individuals (48.4%) survived over the study period (Table 1 and Figure 1).

**Figure 2:**
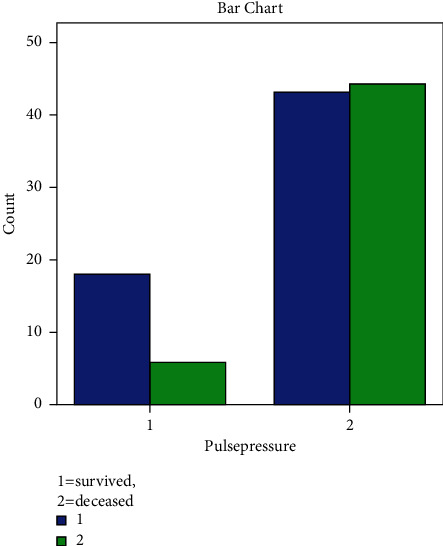
Pulse pressure and mortality. Pulse pressure 1 means less than 60 mmHg, and pulse pressure 2 means widen or more than equal to 61 mmHg.

Table 2 and Figure 2 show that larger gaps in pulse pressure are associated with a higher mortality rate (39.6 percent) compared to normal or small gaps in pulse pressure, which have a mortality rate of 16.2 percent (p 0.05). The odds ratio (OR) of 3 with a 95% confidence range indicates that patients with higher pulse pressure are three times more likely to die. The confidence interval (CI) of 1,112−8,471 suggests that between 1,112 and 8,471 people with acute stroke hemorrhage did not survive.

## 4. Discussion

Pulse pressure is the difference between systolic and diastolic blood pressure. It reflects factors such as stroke volume, arterial compliance, blood velocity, and the distance between the reflexive point and the heart. Research suggests that higher pulse pressure is associated with an increased risk of cardiovascular diseases, including myocardial infarction, stroke, and congestive heart failure [9]. Based on research conducted by Hägg-Holmberg et al. in 2019, there is a direct correlation between pulse pressure and the risk of stroke in the general population [10].

Our findings indicate that individuals with a poor prognosis had higher pulse pressure levels (74.2 mmHg) compared with those who survived. Similarly, Chang et al. discovered that individuals with a bad prognosis had higher pulse pressure levels (68.5 16.4 mm Hg) than those with an excellent prognosis (65.4 12.4 mm Hg) as well [4].

### 4.1. Relationship between Pulse Pressure and Patient Mortality

According to our research, there is a significant connection between pulse pressure and mortality rates in patients with acute hemorrhagic stroke. The odds ratio value of 3 indicates that those with an elevated pulse pressure are three times more likely to experience a fatal outcome than those with a normal or reduced pulse pressure. The results of this study align with Ayyagari et al.'s 2014 research, which emphasized the lasting impact of dilated pulse pressure. Ayyagari concluded that pulse pressure dilation is a significant indicator of an increased stroke risk [11]. Based on the research conducted by Vemmos et al. [12], it was found that a rise in pulse pressure within 24 hours is strongly associated with a higher risk of mortality within a year (with a 95% confidence interval of 1.04–1.86 and a *P* value of 0.028) [12].

Recent findings suggest that a higher pulse pressure during the initial phase of a stroke is associated with a 39% greater chance of mortality within a year. This finding is consistent with the study conducted by Chang and his team in 2017, indicating a significant impact of pulse pressure on mortality and the highest probability of death, even after accounting for other factors. Chang argues that increased pulse pressure is an independent predictor of mortality among ICH patients [4].

A study conducted by Liu et al. in 2020 found similar results to the ones presented here. They concluded that pulse pressure is a significant predictor of mortality in hospitalized ICH patients. Patients with pulse pressures over 101 had 11.0 percent case fatality rate, while those with pulse pressures below 60 had a much lower rate of 1.4 percent [13]. Numerous studies have reported similar findings indicating a correlation between pulse pressure and the mortality rate of patients with acute stroke hemorrhage [14, 15].

According to a study by Mustanoja et al. in 2018, there was no correlation between pulse pressure and mortality in patients with intracerebral hemorrhage (ICH). However, a systolic blood pressure of 160 mmHg during the acute phase was found to be independently associated with higher mortality rates. In addition, at the time of hospital admission, a majority of patients had elevated blood pressure, with 70% having higher systolic blood pressure levels that were linked to increased short-term mortality and long-term mortality. Patients with the highest systolic blood pressure levels had shorter lifespans, highlighting the significance of this measure as a predictor for mortality. A systolic blood pressure of 160 mmHg was found to be associated with both short-term mortality and long-term mortality, while a mean arterial pressure (MAP) of 115 mmHg was linked to long-term mortality. On the other hand, diastolic blood pressure at the time of admission was normal and not independently related to death. Furthermore, there was no observed connection between pulse pressure and mortality. Another study by Mustanoja and Tang found that a pulse pressure of 50 mm Hg was linked to an unfavorable outcome (*P* 0.0001) [8, 16].

Studies on the relevance of pulse pressure in acute strokes have yielded conflicting results. Various factors can lead to an increase in blood pressure among patients with intracerebral hemorrhage (ICH), such as stress, pain, elevated intracranial pressure, reduced autoregulation, heightened systemic vascular resistance, and cerebral endothelial injury, particularly in younger individuals [16].

It is currently unclear whether there is a link between dilated pulse pressure and mortality and how dynamic changes in the acute phase process relate to the chronic characteristics of systemic atherosclerosis. During the acute phase, reduced vascular compliance can cause an increase in pulse pressure to maintain arterial blood flow. This increase in pulse pressure is a dynamic process that may indicate a higher stroke volume or systemic resistance, which can lead to greater bleeding volume and a worse functional outcome [4].

One possible factor is a malfunction in the body's ability to regulate blood flow, which can cause the need to maintain a healthy stroke volume to increase. In cases where this regulation is impaired due to intracerebral hemorrhage (ICH), the pulse pressure may rise to ensure that the brain receives adequate blood flow, resulting in a higher stroke volume and dilated pulse pressure due to an increase in systolic blood pressure. Therefore, it is thought that a sudden and drastic drop in systolic blood pressure, especially in cases of larger ICH bleeding volumes, can decrease cerebral perfusion pressure [4]. However, once the patient is medically and neurologically stable, the reduction in blood pressure can start. On the other hand, an early reduction in mean arterial pressure may be related to ischemic brain injury, as the perihematomal area of an ischemic hematoma is at a risk for ischemia [17].

When there is an increase in pulse pressure, there may also be a higher risk of cerebral edema. The exact reason why pulse pressure is linked to vasogenic edema is still unclear, but previous studies suggest that it may be due to a breach in the blood-brain barrier. This type of edema occurs when there is a breakdown in the barrier that separates blood vessels from brain tissue, allowing fluid and proteins such as albumin to leak into the brain. This can cause swelling and pressure inside the skull. To prevent this from happening, the blood vessels in the brain are uniquely designed to keep harmful substances out. Brain endothelial cells create tight junctions, while astrocytes and pericytes form the blood-brain barrier. However, if the blood-brain barrier is damaged, vasogenic edema can occur. This can happen as a result of brain injury, which can cause mitochondrial failure, excitotoxicity, and oxidative stress. These events can damage the cells that make up the blood-brain barrier, leading to a breach and the development of edema [18].

Moreover, long-term high blood pressure can lead to a gradual decline in the flexibility of blood vessels, causing an increase in pulse pressure and a decrease in diastolic blood pressure. If widened pulse pressure is a symptom of this chronic vascular disease, it is wise to prioritize diagnostic measures and prevention of intracerebral hemorrhage [4].

### 4.2. Limitations of the Research

It is important to highlight that we did not measure intracranial pressure, which is a direct indicator of brain pressure, nor did we record any ICH clots, which are known to increase intracranial pressure and lead to high mortality. We also did not record the usage of anticoagulant and antiplatelet medications. Both types of medication speed up the development of an ICH clot more.

## 5. Conclusions

Research has shown a correlation between wider gaps in pulse pressure and increased mortality rates, with gaps of larger magnitude tripling the chances of death.

## Figures and Tables

**Table 1:** Characteristics and outcomes of intracerebral hemorrhage patients, 2016–2019.

	Total (*N* = 111)	Outcome		*P* value
		Survived (*n* = 61)	Deceased (*n* = 50)	
Mean age, *y*	54.81 + 12.865	53.57 ± 14.51	55.94 ± 10	0.367
Female sex	46 (45.1%)	29 (61.7%)	18 (38.3%)	0.221
Stroke risk factors				
Hypertension		40 (54.1%)	34 (45.9%)	0.670
Diabetes mellitus		39 (53.1%)	33 (45.2%)	0.267
Characteristics on admission				
GCS	10.50 ± 3.949	12.34 ± 3.36	8.36 ± 3.49	*P* ≤ 0.001^*∗*^
Mean HR	88.62 ± 16.38	84.74 ± 12.31	93.28 ± 23.9	0.358
SBPmax	185.95 ± 34.261	177.69 ± 29.010	196.04 ± 37.625	0.496
DBPmax	105.3 ± 18.361	103 ± 17.231	108.10 ± 19.460	0.380
MAPmax	132.15 ± 21.772	127.87 ± 19.653	137.38 ± 23.247	0.075
Pulse pressure	80.625 ± 25.327	74.67 ± 20.49	87.94 ± 28.58	0.026^*∗*^
Lab findings				
Mean glucose	148.63 ± 55.36	139.13 ± 41.392	167.90 ± 73.479	0.267
Mean leucocyte	13872.55 ± 9248.689	11468.856 ± 3815.431	21272 ± 2442	0.457
Mean platelet	270960 ± 79160	258786.89 ± 59951.957	280900 ± 92247.062	0.279
Mean creatinine	0.7 ± 0.09	1.0 ± 0.6	1.478 ± 0.97	0.224

^∗^54.81 ± 12.865

**Table 2:** Relation between admission PP levels and mortality in acute stroke hemorrhage patients.

PP, mmHg	Survive	Deceased	OR crude (95% CI)	OR adjusted (95% CI)
1 (normal, narrow), less <60 mmHg	18 (75%)	6 (25%)	3.07 (1.11–8.47)^*∗*^	2.97 (1.06–8.29)^*∗*^
2 (widen), ≥60 mmHg	43 (49.4%)	44 (50.6%)		

^*∗*^Sig *P* value <0.05.

## Data Availability

The data are deposited in https://doi.org/10.5281/zenodo.7971628.
